# Quadriceps Neuromuscular Impairments after Arthroscopic Knee Surgery: Comparison between Procedures

**DOI:** 10.3390/jcm8111881

**Published:** 2019-11-05

**Authors:** Nicola C. Casartelli, Julia F. Item-Glatthorn, Bernd Friesenbichler, Mario Bizzini, Gian M. Salzmann, Nicola A. Maffiuletti

**Affiliations:** 1 Human Performance Lab, Schulthess Clinic, 8008 Zurich, Switzerland; julia.item@kws.ch (J.F.I.-G.); bernd.friesenbichler@kws.ch (B.F.); mario.bizzini@kws.ch (M.B.); nicola.maffiuletti@kws.ch (N.A.M.); 2 Laboratory of Exercise and Health, ETH Zurich, 8603 Schwerzenbach, Switzerland; 3 Department of Orthopaedic Surgery, Schulthess Clinic, 8008 Zurich, Switzerland; gian.salzmann@kws.ch

**Keywords:** knee arthroscopic surgery, anterior cruciate ligament, meniscus, quadriceps, muscle, strength, atrophy, activation

## Abstract

Quadriceps neuromuscular function remains impaired in the short- and long-term following knee arthroscopy for meniscal surgery and/or anterior cruciate ligament (ACL) reconstruction. The aim of this study was to compare quadriceps neuromuscular impairments in patients following meniscal surgery with and without ACL reconstruction. Thirty patients were tested six months after meniscal surgery with (*n* = 15) and without (*n* = 15) ACL reconstruction. We bilaterally assessed knee extension maximal voluntary contraction (MVC) torque using dynamometry, vastus lateralis thickness using ultrasound, quadriceps voluntary activation and evoked knee extension torque with transcutaneous electrical stimulation. Patient-reported outcomes were evaluated with the Knee Injury and Osteoarthritis Outcome Score (KOOS). Compared with meniscus patients, ACL patients demonstrated larger asymmetries in MVC torque (15% vs. 5%, *p* = 0.049) and vastus lateralis thickness (6% vs. 0%, *p* = 0.021). In ACL patients, asymmetries in MVC torque correlated with asymmetries in evoked torque (*r* = 0.622, *p* = 0.013). In meniscus patients, asymmetries in muscle activation correlated with KOOS quality of life (*r* = 0.619, *p* = 0.018). Patients demonstrated persistent quadriceps muscle weakness six months after ACL reconstruction, but not after isolated meniscal surgery. Quantitative and/or qualitative muscular changes likely underlie quadriceps muscle weakness in ACL patients, whereas activation failure is associated with poor quality of life in some meniscus patients.

## 1. Introduction

Arthroscopic surgery of the knee joint is routinely used for the management of young adults with knee injuries, such as meniscal and/or anterior cruciate ligament (ACL) tears. Isolated ACL injuries are however uncommon [1]. In fact, most of the patients present with other concomitant knee lesions, such as meniscal injuries, which need to be addressed during knee surgery besides ACL reconstruction. Impairments in quadriceps neuromuscular function were reported to persist at short- and long-term follow-ups after ACL surgery, however without differentiating between patients who had isolated ACL reconstruction, and patients who had an associated meniscal treatment [2,3]. In addition, short- and long-term impairments in quadriceps function were observed following isolated arthroscopic meniscal procedures [4,5]. Regardless of knee injury and associated surgical procedures, a quick recovery of quadriceps neuromuscular function through specific and effective rehabilitation protocols is of concern in this population of young and active patients. Poor neuromuscular function may indeed prevent patients from participating in sports activities in the short-term [6], while it may contribute to the development of knee osteoarthritis on a long-term perspective [7]. The magnitude and origin of neuromuscular impairments seem however to differ among patients after arthroscopic knee surgery, depending on the injured knee structures (meniscus and/or ACL) and the respective surgical procedures. Indeed, patients with ACL injuries are subjected to larger unloading conditions than patients with isolated meniscal lesions, both before surgery—due to the larger physical dysfunction associated with ACL rupture/tear—and during postoperative rehabilitation due to the invasiveness of the surgical procedures.

Most of the patients following arthroscopic knee surgery demonstrate an impaired ability to develop maximal voluntary quadriceps strength, which can be due to impairments originating at the neural and/or muscular level [8]. Neural impairments refer to the patients’ inability to voluntarily activate their muscles (activation failure), and seem to mainly underlie quadriceps weakness after isolated meniscal surgeries [5,9]. Activation failure, which is also referred to as arthrogenic muscle inhibition [10], can be caused by general knee symptoms experienced by patients as well as knee pain and/or fear of pain during testing. In contrast, muscular impairments refer to quantitative (i.e., muscle atrophy) and/or qualitative alterations (e.g., impaired muscle contractility), which seem to mainly explain quadriceps weakness after ACL reconstruction [11,12].

A better understanding of the magnitude and origin of neuromuscular impairments after arthroscopic knee surgery would potentially improve the effectiveness of rehabilitation protocols, and reduce the risk of persistent quadriceps weakness at long-term follow-ups. In addition, the comparison between patients who had ACL reconstruction with meniscal treatment, and matched patients who had comparable but isolated meniscal surgery, would reveal the effect of ACL reconstruction alone on quadriceps neuromuscular impairments following arthroscopy knee surgery. Thus, the aims of this study were (i) to compare quadriceps neuromuscular function at short-term follow-up in patients after knee arthroscopic surgery for meniscal treatment with vs. without ACL reconstruction, and (ii) to investigate potential associations between neuromuscular impairments and patient-reported symptoms, function and quality of life. Our hypotheses are that patients after meniscal and ACL treatment will present larger quadriceps neuromuscular impairments compared with patients who had isolated meniscal treatment, and that quadriceps neuromuscular impairments will be associated with patient-reported outcomes.

## 2. Materials and Methods

### 2.1. Study Design and Patients

A total of 30 patients who had knee arthroscopy for the management of acute knee injuries were evaluated at a mean (± SD) of 6 ± 1 months after surgery. At this time point, all patients had completed postoperative rehabilitation, and the short-term effect of the two procedures and associated rehabilitation could be evaluated thus reducing the influence of other external factors. Fifteen patients received knee arthroscopy for the treatment of isolated meniscal lesions (M patients) while 15 patients had knee arthroscopy for ACL rupture with associated meniscal lesions (ACL + M patients). All patients had no prior surgery to the lower limbs (except arthroscopy in the operated knee), and symptoms or signs referable to overt cardiorespiratory, orthopaedic, neurological or general disease, which could have affected quadriceps neuromuscular function. The study was conducted according to the Declaration of Helsinki and the protocol was approved by the Ethics Committee of the Canton of Zurich, Switzerland. All patients signed an informed consent before participating in the study.

### 2.2. Surgical Procedures and Rehabilitation

The procedure for ACL reconstruction consisted of the use of a hamstring graft, while different procedures or combination of procedures were used for the treatment of meniscal lesions (i.e., meniscectomy, meniscal repair, meniscectomy and repair, debridement). Tourniquet pressures between 300 and 350 mmHg were consistently used during all arthroscopic surgeries. During the postoperative hospital stay, the focus of rehabilitation was set on knee range of motion (limited passive knee flexion depending on the surgical procedure), quadriceps control and pain/swelling management. Weight-bearing restrictions were procedure-dependent, ranging from a few weeks (M patients) to 6–8 weeks (ACL + M patients). After discharge, all patients had physical therapy either in our institution or in external rehabilitation facilities but respecting the same postoperative guidelines. Patients in the M group had 6 to 9 physical therapy sessions during a period of six weeks. Patients in the ACL + M group had 29 to 36 physical therapy sessions during a period of 24 weeks. All patients followed a progressive program, which included strengthening, proprioception, coordination and cardiovascular exercises. The strengthening program included exercises for the lower extremity muscles (quadriceps, hamstrings, hip and calf muscles). Knee flexion during weight-bearing exercises (e.g., squats, lunges) was limited to 60° in the first 4–6 weeks after surgery. The exercise intensity ranged between 65–80% of one repetition maximum and the volume was three sets of 12–20 repetitions. Proprioception and coordination exercises included moderate intensity tasks focusing on neuromuscular control of the operated knee such as single-leg balance, and static and dynamic stabilization drills on stable and unstable surfaces for 10 min/session. Cardiovascular exercises included cycling at light-to-moderate intensity for 10 min/session. Within each therapy session, exercise volume was comparable between the two patients’ groups.

### 2.3. Experimental Procedure

Quadriceps neuromuscular function of the operated and non-operated knee was evaluated during a 60-min testing session. Patients were first asked to complete a knee-specific questionnaire to assess knee pain, symptoms and function during activities of daily living (ADL) and sports, as well as quality of life. Then, muscle thickness of the vastus lateralis—as a surrogate of the size of the entire quadriceps muscle [13]—was evaluated at rest using ultrasound imaging. Finally, knee extension torque was recorded during voluntary and/or stimulated contractions to assess maximal voluntary contraction (MVC) torque (as a marker of muscle strength), activation (as a marker of neural function) and evoked torque (as a marker of muscle contractile function). The non-operated knee was always tested first, and a rest period of 5 min was provided before switching to the contralateral operated knee.

### 2.4. Self-Reported Questionnaires

The Knee Injury and Osteoarthritis Outcome Score (KOOS) questionnaire was used [14]. This questionnaire consists of 42 questions with five possible answers. The questions are divided into five subscales: pain (9 questions), symptoms (7 questions), ADL (17 questions), sport (5 questions), and quality of life (4 questions). Each question is scored from 0 to 4, where 0 indicates the highest impairment. The scores are added to produce a single score for each subscale that ranges from 0 to 100, where 0 indicates the highest impairment.

### 2.5. Neuromuscular Parameters

**Figure 1:**
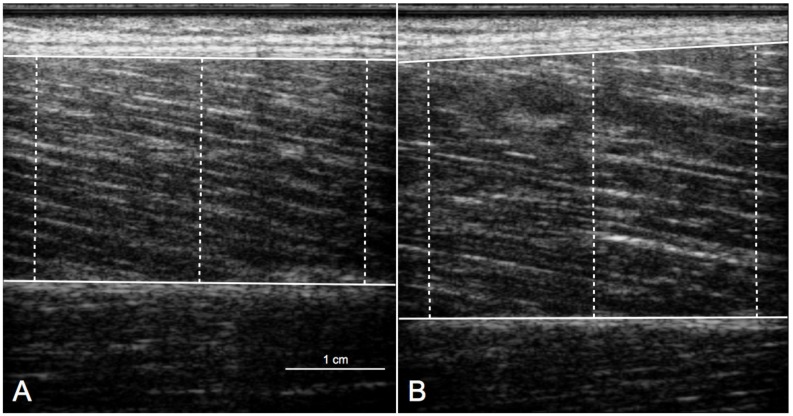
Representative recordings of longitudinal ultrasonographic images of the vastus lateralis muscle on the involved (**A**) and uninvolved side (**B**). The horizontal solid lines represent the superficial (upper line) and deep (lower line) aponeuroses. The vertical dotted lines represent the muscle thickness measured at three different spots (left, middle, right). This patient had anterior cruciate ligament reconstruction with meniscal surgery and showed a −15% side-to-side asymmetry in vastus lateralis thickness.

Muscle thickness of the vastus lateralis was measured using B-mode ultrasound with a linear-array probe (MyLab 25, Esaote, Florence, Italy). Patients were seated on the edge of a treatment table with both hips and knees flexed at 90° and both feet in full contact with the ground. Patients were asked to completely relax the quadriceps muscle during the evaluation. Longitudinal ultrasonic images (width: 3.8 cm; depth: 4 to 6 cm) were recorded at 50% of femur length over the lateral aspect of the vastus lateralis muscle [15,16]. Depending on fascicle visibility, probe frequencies were adjusted between 10 and 15 MHz. A total of three images were taken. Muscle thickness was measured offline by a single experienced investigator using an image-editing software (ImageJ 1.36b, National Institute of Health, Bethesda, MD, USA). The distance between the superficial and deep aponeurosis of the vastus lateralis muscle was measured at three different image spots (left, middle, right). The average muscle thickness of the three spots and images was retained (Figure 1).

**Figure 2:**
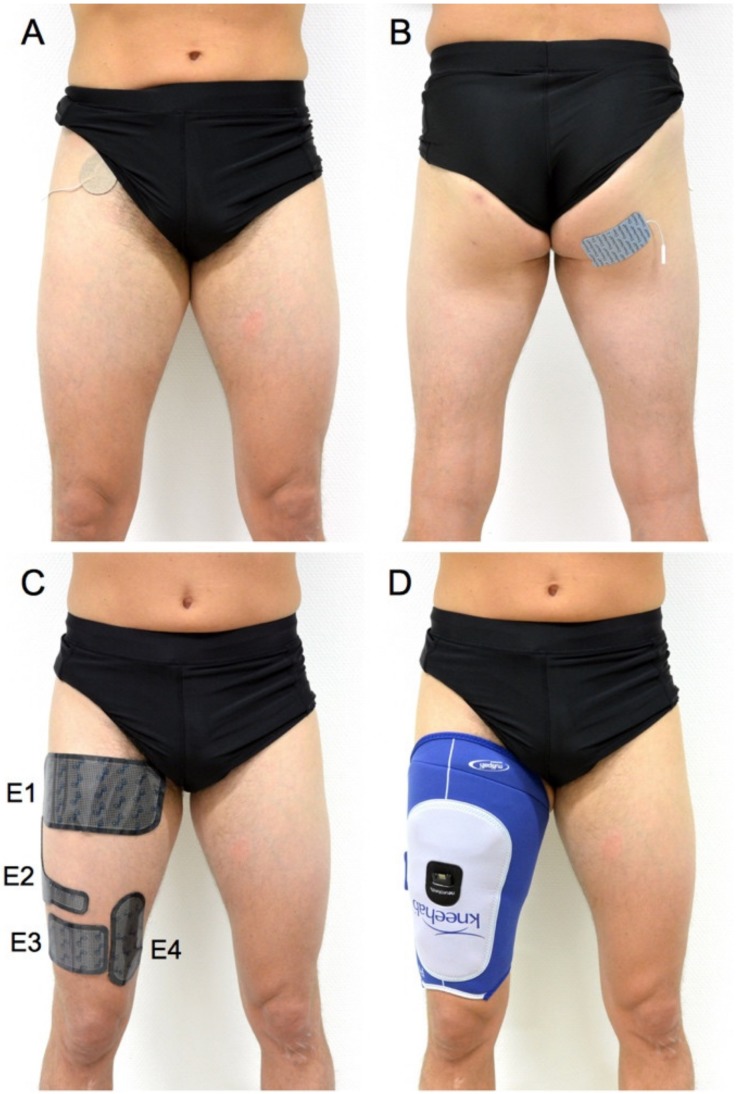
Representation of the two electrode configurations used for electrical stimulation. For the femoral triangle-gluteal fold configuration, a circular electrode was positioned over the femoral triangle (**A**) and a rectangular electrode on the gluteal fold (**B**). For the muscle belly configuration, four electrodes of different shape and size (**C**) were integrated into a garment (**D**) and covered most of the quadriceps muscle bellies. E1, electrode 1; E2, electrode 2; E3, electrode 3; E4, electrode 4.

Knee extension torque was measured using dynamometry (Biodex System 4, Biodex Medical Systems, Shirley, NY, USA). A high-voltage (maximal voltage: 400 V), constant-current stimulator (Model DS7AH, Digitimer Ltd., Hertfordshire, UK) was also used to evoke standardized responses, both superimposed to the MVC (to assess activation level) and at rest (to assess evoked torque). Surface electrodes were either positioned with a femoral triangle-gluteal fold configuration or over the quadriceps muscle bellies. The first configuration consisted of a circular (diameter: 5.08 cm) electrode (American Imex, Irvine, CA, USA) positioned over the femoral triangle to stimulate the femoral nerve, and a rectangular (5 x 10 cm) electrode (Compex, Ecublens, Switzerland) positioned on the gluteal fold (Figure 2A,B). The second configuration consisted of four electrodes integrated into a garment (Kneehab XP, Bio-Medical Research Ltd., Galway, Ireland) (Figure 2C,D). The size of the electrodes was the following: 10 × 20 cm (electrode 1), 3 × 18 cm (electrode 2), 10 × 7.5 cm (electrode 3) and 7 × 14 cm (electrode 4). These two electrode configurations were shown to provide a comparable evaluation of activation and evoked torque [17,18]. Patients were seated on the dynamometer chair with the hips and knees flexed at 80° and 70°, respectively. The dynamometer rotation axis was aligned with the rotation center of the knee joint (i.e., lateral femoral condyle) and the dynamometer lever arm was strapped 2 to 3 cm above the lateral malleolus. Pelvis and trunk were secured to the dynamometer chair with straps. The mass of the tested limb was systematically measured to correct for gravity.

Patients were first familiarized with electrical stimulation; the current intensity of single 1-ms rectangular pulses was progressively increased in 10-mA steps (starting from 0 mA) every 3 to 5 s. Maximal current intensity was determined as the current level at which the evoked torque did not further increase despite increasing current intensity, indicating full quadriceps recruitment [13]. Then, patients underwent a standardized warm-up protocol consisting of 6 to 8 submaximal voluntary contractions and 1 MVC. Following the warm-up, patients completed 3 MVC trials separated by approximately 30 s. They were instructed to maximally contract their quadriceps for 5 s, with a progressive build-up of force. Supramaximal paired stimuli (110–120% of maximal current intensity; 100 Hz) were delivered 1–2 s after contraction onset (to evoke a superimposed twitch), and 2 s after the end of contraction (to evoke a potentiated resting twitch). Standardized verbal encouragements and visual feedback were consistently provided to the patients.

The torque signal was fed directly from the dynamometer into a 16-bit A/D converter (Model MP150, Biopac Systems, Goleta, CA, USA), then into a computer with a sampling frequency of 1 kHz using the AcqKnowledge software (Biopac Systems, Goleta, CA, USA). MVC torque was measured as the peak torque normalized to body mass attained before or after the superimposed twitch. Activation level was calculated using the following formula: [100 − (superimposed twitch torque / potentiated twitch torque) × 100] [19]. Evoked torque was assessed as the potentiated twitch peak torque normalized to body mass. For all the variables, the average of the three trials was retained.

### 2.6. Statistics

Normal distribution of data was assessed using Shapiro–Wilk tests. Data are presented as number, mean ± SD or median with 25th to 75th percentiles. For all neuromuscular parameters (MVC torque, vastus lateralis thickness, activation level and evoked torque), percent side-to-side asymmetries were calculated as [(operated knee / non-operated knee) x 100] - 100. Two-tailed unpaired *t* tests and relative risks with Fisher’s Exact tests or Chi-square tests were used to compare patient characteristics and meniscal procedures between groups (ACL + M vs. M). Differences in neuromuscular parameters between knees (operated vs. non-operated) were evaluated using two-tailed paired *t* tests, and the corresponding differences in side-to-side asymmetries between groups (ACL + M vs. M) were evaluated using two-tailed unpaired *t* tests. Differences in KOOS subscale scores between groups (ACL + M vs. M) were evaluated using Mann-Whitney tests. Associations between neuromuscular parameters and KOOS subscale scores were evaluated with Pearson and Spearman correlation coefficients. Statistical analyses were performed using PASW Statistics 18.0 (SPSS Inc., Chicago, IL, USA). Significance level was set at *p* < 0.05.

## 3. Results

Patients’ characteristics and the different types of surgical procedures for meniscal treatment are reported in Table 1 and Table 2, respectively. No significant differences were observed between the two groups. Neuromuscular parameters are reported in Table 3. In ACL + M patients, significant side-to-side asymmetries were found for MVC torque (2.85 vs. 3.40 Nm/kg, -15%, *p* = 0.001), vastus lateralis thickness (2.47 vs. 2.64 cm, -6%, *p* = 0.009) and evoked torque (0.97 vs. 1.06 Nm/kg, −8%, *p* = 0.014). For M patients, no significant side-to-side asymmetry was observed for any neuromuscular parameter. Significant differences in side-to-side asymmetries between ACL + M and M patients were found for MVC torque (−15% vs. −5%, *p* = 0.049) and vastus lateralis thickness (−6% vs. 0%, *p* = 0.021). KOOS subscale scores are reported in Table 4. Compared with M patients, ACL + M patients reported lower KOOS scores for pain (89 vs. 94 points, *p* = 0.020), symptoms (83 vs. 91 points, *p* = 0.014) and quality of life (63 vs. 72 points, *p* = 0.042).

**Figure 3:**
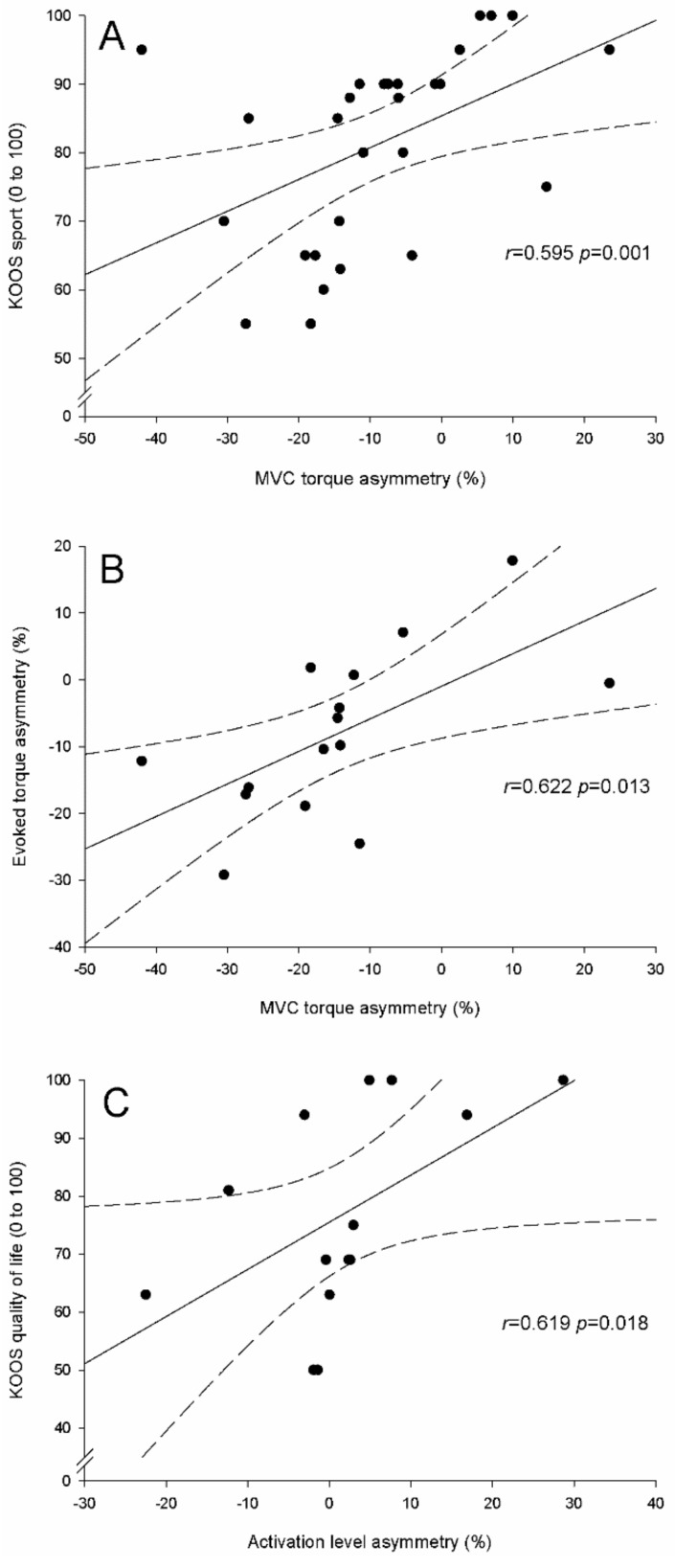
Correlations between quadriceps neuromuscular parameters and patients’ characteristics, function or quality of life in (**A**) all patients, (**B**) ACL + M patients, and (**C**) M patients. KOOS, Knee Injury and Osteoarthritis Outcome Score; MVC, maximal voluntary contraction; VL, vastus lateralis. M, isolated meniscal surgery; ACL + M, anterior cruciate ligament reconstruction with meniscal surgery.

Considering all patients together, asymmetries in MVC torque were positively correlated with KOOS pain (*r* = 0.385, *p* = 0.043), symptoms (*r* = 0.446, *p* = 0.017), quality of life (*r* = 0.476, *p* = 0.011) and sport (*r* = 0.595, *p* = 0.001) (Figure 3A), as well as with asymmetries in evoked torque (*r* = 0.444, *p* = 0.014). Asymmetries in evoked torque were positively correlated with KOOS sport (*r* = 0.395, *p* = 0.037). For ACL + M patients, asymmetries in MVC torque and evoked torque correlated positively (*r* = 0.622, *p* = 0.013) (Figure 3B). For M patients, activation level and KOOS quality of life were positively correlated (*r* = 0.619, *p* = 0.018) (Figure 3C).

## 4. Discussion

The magnitude of both quadriceps muscle weakness and vastus lateralis atrophy was greater six months after arthroscopic ACL and meniscal surgery compared to isolated meniscal surgery. Regardless of surgical procedure, strength deficits were larger in patients with higher levels of self-reported knee pain and symptoms, as well as lower knee function during sports and quality of life. In patients who had combined ACL and meniscal surgery, strength deficits were associated with poor muscle contractile function, as witnessed by evoked torque results. In contrast, quadriceps activation failure was associated with lower quality of life in patients after isolated meniscal surgery.

### 4.1. Strengths and Limitations

Quadriceps neuromuscular function was already assessed in patients after ACL or meniscal arthroscopic procedures, but always separately [3,5,9,12,20]. To the best of our knowledge, this is the first study that directly compared the magnitude and origin of neuromuscular impairments after knee arthroscopic surgery between these two groups of patients. Moreover, the uniqueness of our present study was to investigate the effect of ACL reconstruction alone, which mostly needs simultaneous meniscal treatment, on postoperative quadriceps neuromuscular impairments, by using patients with isolated meniscal surgery as controls. The main limitation of the present cross-sectional study is that quadriceps neuromuscular function was not evaluated preoperatively. Thus, we cannot determine whether the impairments observed six months after surgery were already present before surgery, or rather they developed during the postoperative time period. In addition, another limitation of this study is that quadriceps activation level was estimated by comparing the operated to the contralateral, asymptomatic knee. Because patients after knee injury/surgery frequently present with bilateral quadriceps activation failure [21], it would have been more judicious to include a group of healthy controls. Moreover, different weight-bearing restrictions and total physical therapy volumes were experienced by the two groups of patients, with patients after ACL and meniscus surgery with longer weight-bearing restrictions and higher total exercise volume. These discrepancies in rehabilitation protocols are mainly due to the different injury and treatment of various knee structures in the two patients’ groups, which logically required different recovery and unloading periods. Even if these discrepancies might have influenced quadriceps neuromuscular function recovery, they are dictated by the surgical procedures and thus hardly modifiable in the clinical practice.

### 4.2. Quadriceps Muscle Weakness in ACL and Meniscus Patients

Six months after knee arthroscopy, patients who had combined ACL and meniscal surgery still demonstrated significant quadriceps muscle strength deficits (average asymmetry of 15%), contrary to the patients after isolated meniscal surgery (average asymmetry of 5%). Strength asymmetries up to 10% are usually considered to be “normal”, while asymmetries between 10% and 20% should be seen as “probably pathological” [8]. Our findings are in agreement with previous results reported from patients after ACL reconstruction [3], but not with prior studies on patients after partial meniscectomy. Indeed, previously tested meniscus patients demonstrated on average 12% to 21% MVC torque asymmetries six months after surgery [5,20]. Interestingly, asymmetries in muscle strength, but not in other neuromuscular parameters, were strongly associated with several patient-reported outcomes, such as knee pain, symptoms and function during sports, as well as quality of life in the entire group of our patients. These results highlight the fact that their voluntary force-generating capacity is significantly influenced by residual knee symptoms following surgery, regardless of the arthroscopic surgical procedure [22]. In addition, incomplete recovery of quadriceps strength seems to have negative consequences on knee function, especially during sport, and quality of life in young patients [22].

### 4.3. Neuromuscular Impairments in ACL Patients

Deficits in quadriceps muscle mass and/or contractile function seem to be the main factors underlying residual muscle weakness in patients after combined ACL and meniscal surgery. Indeed, muscle thickness and evoked torque showed significant asymmetries between the operated and the non-operated knee. These findings were further strengthened by the observation that strength asymmetry, which ranged between −42% and 24%, was related to the evoked torque asymmetry, which ranged between −29% to 18%. The lack of relationship between strength and thickness asymmetries is probably due to the fact that thickness was only evaluated for a single – even if representative – quadriceps muscle head (i.e., vastus lateralis), but not for the others (i.e., vastus medialis and intermedius, rectus femoris), which might even undergo greater muscle atrophy [12]. In contrast, the evaluation of muscle contractile function by means of the evoked torque provides a good estimation of the entire quadriceps muscle mass [23]. The observed evoked torque asymmetries might also indicate that qualitative besides quantitative alterations, such as fatty degeneration, may affect quadriceps function after ACL reconstruction. As a matter of fact, a series of molecular degenerative processes (e.g., collagen accumulation, increased fibroblast content, reduced satellite cell abundance) have recently been observed in the vastus lateralis of patients early following an ACL injury [24].

### 4.4. Neuromuscular Impairments in Meniscus Patients

Even though patients after isolated meniscal surgery did not show any significant quadriceps neuromuscular impairments, it seems that residual quadriceps activation failure in some patients might have important consequences for their quality of life. In the present study, the average level of quadriceps activation was in a normal range (> 90%) and did not differ between the operated and non-operated knee. Nevertheless, activation asymmetries varied considerably among patients (‒29% to 23%). This indicates that some patients still display quadriceps activation failure six months after isolated meniscal surgery, and interestingly these patients are those who reported low levels of quality of life. It can be speculated that the inability to adequately activate the quadriceps muscle after surgery might prevent some patients to participate in sport activities, especially those they realized before knee injury [22], which may in turn affect their quality of life. This assumption was not fully supported by our results, with the relationship between activation asymmetry and the reported knee function during sports (KOOS sport) being relatively high, though not significant (*r* = 0.413, *p* = 0.142). We suppose that the questionnaire failed to detect some patient-specific impairments during sports, since it only asked on difficulties in performing a series of general sporting tasks (e.g., kneeling, squatting).

## 5. Conclusions

Six months following knee arthroscopy, patients after combined ACL and meniscal surgery, but not patients after isolated meniscal surgery, still demonstrate significant quadriceps strength deficits and atrophy. For patients after combined ACL and meniscal surgery, quantitative and/or qualitative muscular alterations seem to underlie the persistent quadriceps muscle weakness. For some of the patients after isolated meniscal surgery, residual quadriceps activation failure has a possible negative effect on their quality of life. Patients after combined ACL and meniscal surgery may benefit from further strengthening exercises at six months following knee arthroscopy in order to fully recover the quadriceps neuromuscular function, while the few patients with residual quadriceps activation failure after isolated meniscal surgery may benefit for example from neuromuscular/transcutaneous electrical stimulation, cryotherapy or additional proprioception exercises.

## Figures and Tables

**Table 1:** Patient characteristics by group.

	Mean ± SD / Number			Mean Difference / Relative Risk (95% CI)	*p* Value
	All brk (*n* = 30)	M brk (*n* = 15)	ACL + M brk (*n* = 15)		
Gender (women / men)	4 / 26	1 / 14	3 / 12	1.62 (0.81 to 3.28)	0.598
Age (years)	32 ± 9	35 ± 10	29 ± 7	6 (−1 to 13)	0.077
Body mass (kg)	74 ± 10	76 ± 7	73 ± 11	3 (−4 to 10)	0.386
Height (cm)	176 ± 8	178 ± 7	174 ± 9	4 (−2 to 10)	0.190
BMI (kg/m^2^)	24 ± 2	24 ± 2	24 ± 2	0 (−1 to 1)	0.909

BMI, body mass index; SD, standard deviation; CI, confidence interval; M, isolated meniscal surgery; ACL + M, anterior cruciate ligament reconstruction with meniscal surgery.

**Table 2:** Meniscal surgery procedures by group.

	Number			Relative Risk (95% CI)	*p* Value
	All brk (*n* = 30)	M brk (*n* = 15)	ACL + M brk (*n* = 15)		
Partial meniscectomy	17	9	8	0.87 (0.42 to 1.82)	0.713
Meniscal repair	7	3	4	1.22 (0.47 to 3.12)	1.000
Partial meniscectomy and repair	3	2	1	0.72 (0.30 to 1.76)	1.000
Meniscal debridement	3	1	2	1.56 (0.30 to 8.03)	1.000

CI, confidence interval; M, isolated meniscal surgery; ACL + M, anterior cruciate ligament reconstruction with meniscal surgery.

**Table 3:** Quadriceps neuromuscular parameters by knee and group.

	Operated Knee	Non-Operated Knee	*p* Value	Side-to-Side Asymmetry (%)		*p* Value
	Mean ± SD			Mean ± SD	Mean Difference (95%CI)	
**MVC torque (Nm/kg)**						
All (*n* = 30)	3.16 ± 0.61	3.54 ± 0.66	<0.001	−10 ± 14		
*M* (*n* = 15)	3.47 ± 0.49	3.68 ± 0.64	0.052	−5 ±10	10 (0 to 20)	0.049
*ACL + M* (*n* = 15)	2.85 ± 0.57	3.40 ± 0.68	0.001	−15 ± 16		
**Vastus lateralis thickness (cm)**						
All (*n* = 30)	2.53 ± 0.34	2.62 ± 0.33	0.032	−3 ± 8		
*M* (*n* = 15)	2.60 ± 0.25	2.60 ± 0.27	0.953	0 ± 7	6 (1 to 12)	0.021
*ACL + M* (*n* = 15)	2.47 ± 0.41	2.64 ± 0.40	0.009	−6 ± 8		
**Activation level (%)**						
All (*n* = 30)	91 ± 8	92 ± 8	0.654	0 ± 9		
*M* (*n* = 15)	91 ± 9	91 ± 9	0.754	2 ± 11	4 (-3 to 10)	0.292
*ACL + M* (*n* = 15)	91 ± 8	93 ± 7	0.203	−2 ± 6		
**Evoked torque (Nm/kg)**						
All (*n* = 30)	1.10 ± 0.27	1.16 ± 0.28	0.013	−5 ± 12		
*M* (*n* = 15)	1.23 ± 0.20	1.27 ± 0.22	0.329	−2 ± 11	6 (-3 to 15)	0.188
*ACL + M* (*n* = 15)	0.97 ± 0.26	1.06 ± 0.30	0.014	−8 ± 12		

MVC, maximal voluntary contraction; M, isolated meniscal surgery; ACL + M, anterior cruciate ligament reconstruction with meniscal surgery; SD, standard deviation; CI, confidence interval. *p* values are reported for the differences between the operated vs. non-operated knee, and for the differences in side-to-side asymmetry between M and ACL + M patients.

**Table 4:** Patient-reported outcomes by group.

	Median (25th to 75th Percentile)			*p* Value
	All brk (*n* = 28)	M brk (*n* = 14)	ACL + M brk (*n* = 14)	
KOOS pain (0 to 100)	92 (83 to 96)	94 (85 to 100)	89 (78 to 92)	0.020
KOOS symptoms (0 to 100)	89 (79 to 95)	91 (86 to 97)	83 (68 to 90)	0.014
KOOS ADL (0 to 100)	99 (96 to 100)	100 (96 to 100)	99 (95 to 100)	0.645
KOOS sport (0 to 100)	87 (66 to 90)	90 (79 to 91)	75 (62 to 91)	0.087
KOOS quality of life (0 to 100)	69 (52 to 81)	72 (63 to 96)	63 (49 to 75)	0.042

KOOS, Knee Injury and Osteoarthritis Outcome Score; ADL, activities of daily living; M, isolated meniscal surgery; ACL + M, anterior cruciate ligament reconstruction with meniscal surgery. Two patients (one in each group) did not complete the KOOS questionnaire.
